# Effects of Rivaroxaban on Biomarkers of Coagulation and Inflammation: A Post Hoc Analysis of the X-VeRT Trial

**DOI:** 10.1055/s-0040-1701206

**Published:** 2020-01-23

**Authors:** Paulus Kirchhof, Michael D. Ezekowitz, Yanish Purmah, Sonja Schiffer, Isabelle L. Meng, A. John Camm, Stefan H. Hohnloser, Anke Schulz, Melanie Wosnitza, Riccardo Cappato

**Affiliations:** 1 Institute of Cardiovascular Sciences, University of Birmingham, Sandwell and West Birmingham Hospitals NHS Trust and University Hospitals Birmingham NHS Foundation Trust, Birmingham, United Kingdom; 2 Department of Cardiology, University Heart Center Hamburg, Hamburg, Germany; 3 Department of Cardiovascular Medicine, The Sidney Kimmel Medical College, Thomas Jefferson University, and the Lankenau Medical Center, Philadelphia, Pennsylvania, United States; 4 Translational Medicine, Global R&D, Bayer AG, Wuppertal, Germany; 5 Therapeutic Area Cardiovascular, Global Medical Affairs, Bayer AG, Berlin, Germany; 6 Division of Clinical Sciences, St George's, University of London, London, United Kingdom; 7 Division of Clinical Electrophysiology, Department of Cardiology, J. W. Goethe University, Frankfurt, Germany; 8 Clinical Statistics EU, Global Clinical Development, Research and Clinical Sciences Statistics, Bayer AG, Berlin, Germany; 9 Arrhythmia and Electrophysiology Research Center, Humanitas Clinical and Research Center, Rozzano, Italy

**Keywords:** anticoagulants, atrial fibrillation, biomarkers, inflammation, rivaroxaban

## Abstract

**Introduction** This X-VeRT (eXplore the efficacy and safety of once-daily oral riVaroxaban for the prevention of caRdiovascular events in patients with nonvalvular aTrial fibrillation scheduled for cardioversion) substudy evaluated the effects of treatment with rivaroxaban or a vitamin-K antagonist (VKA) on levels of biomarkers of coagulation (D-dimer, thrombin–antithrombin III complex [TAT] and prothrombin fragment [F1.2]) and inflammation (high sensitivity C-reactive protein [hs-CRP] and high-sensitivity interleukin-6 [hs-IL-6]) in patients with atrial fibrillation (AF) who were scheduled for cardioversion and had not received adequate anticoagulation at baseline (defined as, in the 21 days before randomization: no oral anticoagulant; international normalized ratio <2.0 with VKA treatment; or <80% compliance with non-VKA oral anticoagulant treatment).

**Methods** Samples for biomarker analysis were taken at baseline ( *n* = 958) and treatment completion (42 days after cardioversion; *n* = 918). The influence of clinical characteristics on baseline biomarker levels and the effect of treatment on changes in biomarker levels were evaluated using linear and logistic models.

**Results** Baseline levels of some biomarkers were significantly associated with type of AF (D-dimer and hs-IL-6) and with history of congestive heart failure (hs-CRP, D-dimer, and hs-IL-6). Rivaroxaban and VKA treatments were associated with reductions from baseline in levels of D-dimer (−32.3 and −37.6%, respectively), TAT (−28.0 and −23.1%, respectively), hs-CRP (−12.5 and −17.9%, respectively), and hs-IL-6 (−9.2 and −9.8%, respectively). F1.2 levels were reduced from baseline in patients receiving a VKA (−53.0%) but not in those receiving rivaroxaban (2.7%).

**Conclusion** Anticoagulation with rivaroxaban reduced levels of key inflammation and coagulation biomarkers to a similar extent as VKAs, with the exception of F1.2. Further investigation to confirm the value of these biomarkers in patients with AF is merited.

## Introduction

Atrial fibrillation (AF) is the most common cardiac arrhythmia. The prevalence of AF is approximately 3% among adults aged 20 years or older but increases with age to more than 15% among adults aged 80 years or older. 1 2 Pharmacological or electrical cardioversion can be used to restore sinus rhythm rapidly. However, cardioversion is associated with a periprocedural risk of thromboembolic events of up to 9% in patients who have not received anticoagulants. 3 4 5 To minimize this risk, immediate initiation of anticoagulation is recommended in all patients scheduled for cardioversion. 5

AF is associated with a prothrombotic state; elevated levels of the circulating coagulation biomarkers D-dimer, prothrombin fragment 1 + 2 (F1.2), and thrombin–antithrombin III complex (TAT) have been detected in patients with AF. 6 Vitamin-K antagonist (VKA) therapy has been shown to significantly suppress levels of D-dimer, F1.2, and TAT. 7 There is evidence that inflammation is a driver of the prothrombotic state and that it plays a significant role in the pathogenesis of AF. 8 9 10 11 Elevated levels of the inflammation biomarkers high-sensitivity interleukin-6 (hs-IL-6) and high-sensitivity C-reactive protein (hs-CRP) have been observed in patients with AF. 11 12 Inflammatory mediators including ILs are thought to promote arrhythmogenesis as a result of structural and contractile remodeling of the atria and endocardium. 11 There have also been reports of a correlation between inflammatory mediators and the duration of AF and success of cardioversion, suggesting a possible predictive role for these biomarkers. 11 12

Rivaroxaban is a non-VKA oral anticoagulant (NOAC) that directly inhibits factor Xa. There is evidence to suggest that, in addition to its role in coagulation, factor Xa has proinflammatory effects. For example, a reduction in the levels of hs-CRP has been demonstrated in patients with AF who received enoxaparin (an indirect factor Xa inhibitor) as bridging therapy before cardioversion. 13 It is, therefore, possible that inhibition of factor Xa with rivaroxaban may induce anti-inflammatory responses and have a protective effect on the vascular endothelium, in addition to anticoagulant actions. 14 The effects of rivaroxaban on selected biomarkers of coagulation have previously been demonstrated in patients with venous thromboembolism. 15 16 However, the effects of rivaroxaban on a combination of coagulation and inflammation biomarkers in patients with AF undergoing cardioversion have not yet been fully evaluated.

The X-VeRT (eXplore the efficacy and safety of once-daily oral riVaroxaban for the prevention of caRdiovascular events in patients with nonvalvular aTrial fibrillation scheduled for cardioversion) study investigated the efficacy and safety of rivaroxaban compared with a VKA for thromboprophylaxis in patients with non-valvular AF scheduled for cardioversion. Patients with hemodynamically significant mitral valve stenosis or prosthetic heart valves were excluded. 17 18 This substudy of X-VeRT evaluated the effects of rivaroxaban and VKAs on biomarkers of coagulation (D-dimer, TAT, and F1.2) and biomarkers of inflammation (hs-IL-6 and hs-CRP) in the same patient population.

## Materials and Methods

### X-VeRT Study Design

X-VeRT was a prospective, randomized, open-label, parallel-group, active-controlled, multicenter, and phase-IIIb study. 17 18 In brief, patients with hemodynamically stable nonvalvular AF (of more than 48 hours or unknown duration) who were scheduled for electrical or pharmacological cardioversion were randomized in a 2:1 ratio to receive rivaroxaban or a VKA. Patients randomized to receive rivaroxaban were given a once-daily oral dose of 20 mg (reduced to 15 mg in patients with a creatinine clearance of 30–49 mL/min), and those randomized to receive a VKA (warfarin or another VKA based on local standard of care) were treated with the aim of achieving a target international normalized ratio (INR) of 2.5 (range, 2.0–3.0). A parenteral anticoagulant drug could be administered in addition to VKA therapy, especially before cardioversion, until the target INR was reached.

One of two cardioversion strategies was selected before randomization at the discretion of the investigator in accordance with the protocol. 18 Patients treated with an early cardioversion strategy received rivaroxaban or usual VKA therapy for 1 to 5 days before the procedure and 42 days thereafter. Patients treated with a delayed cardioversion strategy received rivaroxaban or a VKA for 3 to 8 weeks before the procedure and 42 days thereafter. 18

### Biomarker Substudy: Objectives and Patient Population

The overall objectives of the biomarker substudy were to evaluate biomarkers of coagulation (D-dimer, TAT, and F1.2) and inflammation (hs-CRP and hs-IL-6) in patients receiving rivaroxaban or a VKA in the X-VeRT study. The specific aims were to (1) evaluate the influence of treatment with rivaroxaban or a VKA on changes in biomarker levels from baseline to the end of treatment; (2) investigate the influence of pretreatment with oral anticoagulants (OACs) and medical history on biomarker levels at baseline; and (3) explore the predictive and prognostic value of the selected biomarkers.

Patients in the modified intention-to-treat (mITT) population of X-VeRT (which excluded patients in whom a left atrial thrombus was detected before cardioversion) were eligible for inclusion in the biomarker substudy. At baseline, samples for biomarker analysis were only taken from patients considered to have had inadequate oral anticoagulation before randomization because, in those patients, biomarker levels were assumed to be the least influenced by prior anticoagulation. These patients included the following: (1) those who had been treated with a VKA in the 21 days before randomization but had an INR of less than 2.0 at any time in that period; (2) those who had been treated with a NOAC in the 21 days before randomization but in whom compliance was less than 80% in that period; and (3) those who had not received OACs in the 21 days before randomization or had never received any OAC prior to the study. Patients were defined as untreated or naive to OACs if they had not taken a VKA or NOAC in the 6 weeks before entering the study.

### Blood Sample Collection

Venous blood samples were taken at the screening visit, and then again at the end of treatment visit (or at the time of early discontinuation of study treatment). Depending on the cardioversion treatment strategy, the end of treatment sample was taken 43 (+4) days (early cardioversion) or 63 to 98 (+4) days (delayed cardioversion) after the first intake of study drug. For the end of treatment sample, patients had to have taken the last dose of study drug within 24 hours of the sampling for biomarkers.

### Biomarker Analysis

A laboratory manual, detailing instructions for the handling and shipment of blood samples, was provided to each site. Blood samples were immediately centrifuged for plasma preparation, frozen, and stored at −20°C or below before being shipped on dry ice for analysis. All biomarkers were analyzed by Covance CLS (Indianapolis, Indiana, United States). Concentrations of the coagulation biomarkers D-dimer, F1.2, and TAT were measured in citrated plasma using immunoturbidimetry for D-dimer (INNOVANCE D-dimer assay kit [intra- and interassay precision 1.5–7.8% and 2.2–7.9% coefficient of variation [CV], respectively; Siemens Healthcare Diagnostics, Newark, Delaware, United States], Siemens BCS, and BCS XP) and enzyme-linked immunosorbent assay (ELISA) for F1.2 and TAT (Enzygnost F1 + 2 kit [intra- and interassay precision 3.6–5.5% and 4.4–11.2% CV, respectively] and Enzygnost TAT kit [intra- and interassay precision 3.1–6.0% and 6.0–9.0% CV, respectively], Siemens Healthcare Diagnostics Products, Marburg, Germany). Reference ranges are less than 0.59 mg/L fibrinogen equivalent units (FEU) for D-dimer, 69 to 229 pmol/L for F1.2 and 1.0 and 4.1 µg/L for TAT.

Concentrations of the inflammation biomarkers hs-CRP and hs-IL-6 were measured in serum or ethylenediaminetetraacetic acid (EDTA) plasma using immunonephelometry for hs-CRP (N hsCRP reagent [Siemens Healthcare Diagnostics, Deerfield, Illinois, United States] and Siemens BN II Nephelometer [intra- and interassay precision 2.3–4.4% and 2.1–5.7% CV, respectively]) and ELISA for hs-IL-6 (Quantikine Human hs-IL-6 Immunoassay kit [intra- and interassay precision 6.9–7.8% and 6.5–9.6% CV, respectively], R&D Systems, Minneapolis, Minnesota, United States). Reference ranges are 0.287 mg/dL or below for hs-CRP and less than 12.50 pg/mL for hs-IL-6.

### Clinical Events

To determine the prognostic/predictive value of the biomarkers, the relationship between biomarker levels and major and nonmajor bleeding events were evaluated. All bleeding events were adjudicated. Major and nonmajor bleeding events were defined according to the International Society on Thrombosis and Hemostasis criteria. 19 The relationship between biomarker levels at baseline and time to cardioversion and whether or not cardioversion was successful was also examined. Cardioversion was considered successful if restoration of normal sinus rhythm was achieved after the first electrical or pharmacological cardioversion during the study.

### Statistical Methods

All analyses were performed using SAS version 9.4 or later versions (SAS Institute Inc., Cary, North Carolina, United States) and R version 3.1.0 or later versions. 20

The number of patients included in this substudy was based on feasibility and clinical judgment rather than on statistical considerations relating to the biomarker identification objectives. Relationships between biomarker levels and variables of interest were analyzed by linear and logistic regression models; a significance *α* level of 0.05 was used for all models. All five biomarkers were characterized by a heavily skewed distribution; therefore, the data were log-transformed prior to regression analysis.

#### Factors Influencing Biomarker Levels at Baseline

Relevant demographic characteristics (out of sex, race, age, and body mass index [BMI]) for the baseline level of each biomarker were identified by means of multivariable linear regression models. Potentially relevant demographic characteristics were selected for each biomarker based on the minimal Akaike information criterion (AIC). The selected demographic characteristics were considered as covariates in linear regression models for the analysis of the relationship between biomarker baseline levels and medical history and prior OAC intake, to control for any influence that they may have had on the studied biomarkers.

#### Relationship between Biomarker Levels at Baseline and Clinical Events during the Study

The prognostic or predictive potential of biomarker levels at baseline was assessed by logistic models for the binary endpoints (bleeding events) and by a linear model for the (log-transformed) time to cardioversion. Relevant covariates were chosen from the pool of potential covariates (demographic characteristics, medical history, cardioversion strategy, prior OAC intake, treatment, and CHADS2 [congestive heart failure (HF), hypertension, age ≥ 75 years, diabetes mellitus, prior stroke, thromboembolism, or transient ischemic attack (TIA)] score) by means of a stepwise AIC forward selection. The resulting base models were then expanded by the set of biomarkers (as main effects or in interaction with treatment), which led to an overall best model for the respective endpoint. This was also accomplished by a stepwise AIC forward selection (i.e., only those biomarkers that led to an expected improvement in endpoint prediction were added to the model).

The AIC-based variable selection that was applied in these analyses is an established method to prefilter relevant variables for an endpoint of interest. 21 Variables that do not improve the prediction accuracy for the respective endpoint are excluded to decrease the number of parameters and, hence, improve the stability of the results in the multivariable analyses.

#### Influence of Treatment and Other Factors on Changes in Biomarker Levels from Baseline to End of Treatment

Biomarker changes from baseline to end of treatment were analyzed and compared between the two treatment arms by linear regression models, adjusted for biomarker baseline levels, and the differences in treatment duration that were induced by the study design. The effects of medical history, prior OAC intake, cardioversion strategy, and cardioversion success on the biomarker changes were investigated by adding the respective information to the models as main effects and in interaction with the treatment.

## Results

### Patients

**Fig. 1:**
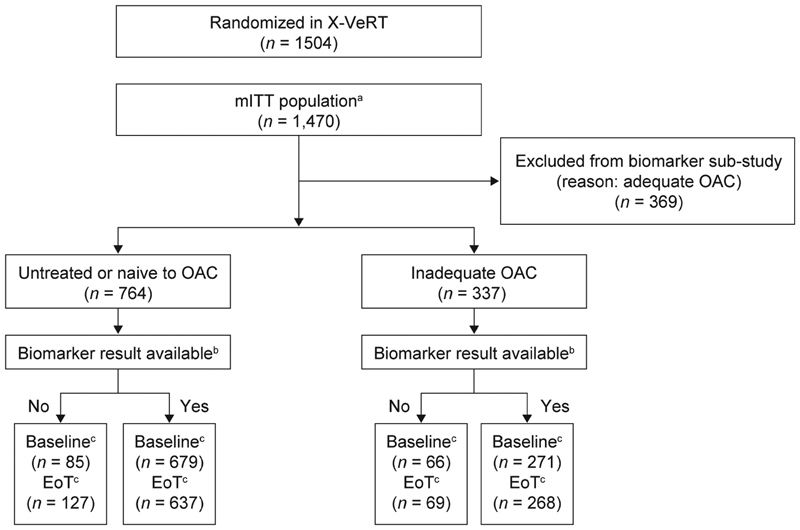
Patient flow and overview of biomarker collection. EoT, end of treatment; hs-CRP, high-sensitivity C-reactive protein; mITT, modified intention-to-treat; OAC, oral anticoagulant. ^a^ Patients in the mITT population in whom a left atrial thrombus was not diagnosed during transesophageal echocardiography performed before the first planned cardioversion. ^b^ Reasons for missing results, for example, specimen not frozen, insufficient quantity, hemolysis. ^c^ Availability of samples based on available hs-CRP measurements; end of treatment samples could still be collected from patients without a baseline sample.

In the X-VeRT study, 1,504 patients in total were randomized to receive either rivaroxaban (1,002 patients; 66.6%) or a VKA (502 patients; 33.4%). 17 The mITT population of 1,470 patients (rivaroxaban group, 978 patients; VKA group, 492 patients) included 1,101 patients who were untreated or naive to OACs or had received inadequate OAC treatment before randomization and were, therefore, eligible for inclusion in the biomarker substudy ( Fig. 1 ). 17 Blood samples for biomarker analysis were collected from a total of 958 patients at baseline and 918 patients at the end of treatment. An overview of the number of measurements available for each biomarker is provided in Supplementary Table S1 , and a comparison of baseline characteristics between the two treatment arms is given in Supplementary Table S2 .

### Factors Influencing Biomarker Levels at Baseline

#### Patient Characteristics

Relevant demographic characteristics (out of sex, race, age, and BMI) for the baseline level of each biomarker are displayed in Table 1 . Overall, only a small fraction of biomarker variability (between 1.6% [TAT] and 11.2% [D-dimer]) was explained by patient characteristics. The relevant demographic variables for each biomarker were considered as covariates for the following analyses of pretreatment and medical history.

**Table 1:** Proportion of variability in biomarker baseline levels explained by demographic variables a

Biomarker	Sex	Race	Age	BMI	Explained variability (%)
hs-CRP	x		x	x	5.6
D-dimer			x	x	11.2
hs-IL-6			x	x	9.2
F1.2	x		x		1.7
TAT		x	x		1.6

Abbreviations: BMI, body mass index; F1.2, prothrombin fragment 1 + 2; hs-CRP, high-sensitivity C-reactive protein; hs-IL-6, high-sensitivity interleukin-6; TAT, thrombin–anti-thrombin III complex.

a Relevant set of demographic variables for each biomarker (marked by an “x”) was selected by AIC (Akaike's information criterion) optimization.

#### Pretreatment with OACs

**Fig. 2:**
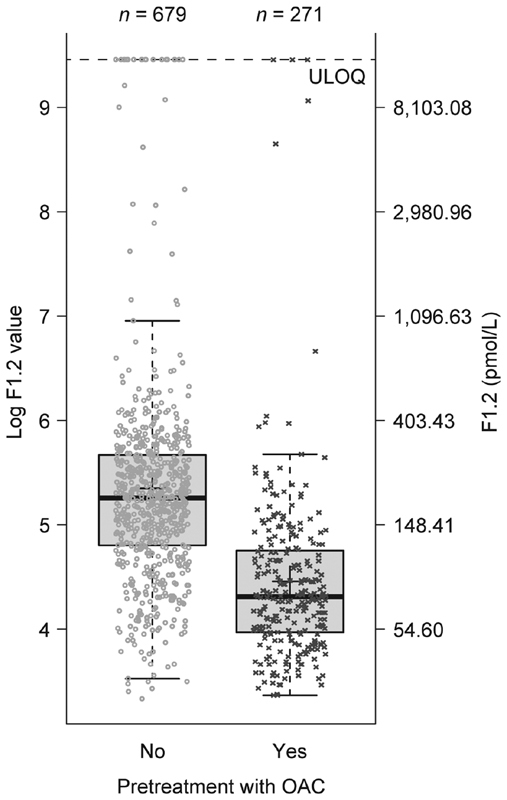
Influence of pretreatment with OACs on F1.2 levels at baseline. F1.2, prothrombin fragment 1 + 2; OAC, oral anticoagulant; ULOQ, upper limit of quantification. Note: Upper lines of the box denote the upper quartiles, midlines denote the medians and lower lines denote the lower quartiles; crosses denote the geometric means, and upper and lower lines denote maximum and minimum values, excluding outliers (i.e., values that are >1.5 times the interquartile range further apart from the box).

In patients who had received pretreatment with OACs, baseline levels of F1.2, D-dimer and TAT were lower than in patients who were untreated or naive to OACs (F1.2: 87.23 vs. 214.99 pmol/L, *p* < 0.001; D-dimer: 0.20 vs. 0.40 mg/L FEU, *p* < 0.001; and TAT: 4.48 vs. 5.71 ug/L, *p* = 0.003). Pretreatment with OACs did not significantly influence baseline levels of hs-CRP or hs-IL-6 ( Fig. 2 [F1.2 only] and Supplementary Table S3 ).

#### Medical History

**Fig. 3:**
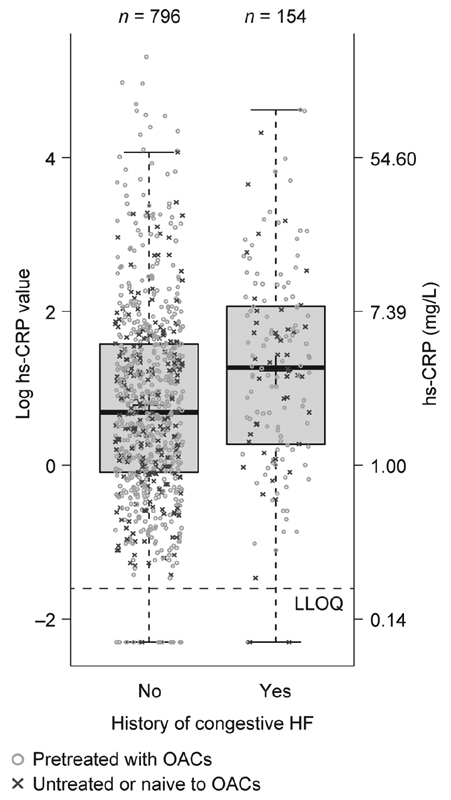
Influence of a history of congestive HF on hs-CRP levels at baseline. HF, heart failure; hs-CRP, high-sensitivity C-reactive protein; LLOQ, lower limit of quantification; OAC, oral anticoagulant. Note: Upper lines of the box denote the upper quartiles, midlines denote the medians and lower lines denote the lower quartiles; crosses denote the geometric means, and upper and lower lines denote maximum and minimum values, excluding outliers (i.e., values that are >1.5 times the interquartile range further apart from the box).

In patients with a history of congestive HF, mean baseline levels of hs-CRP, D-dimer, and hs-IL-6 were significantly higher than in patients with no history of congestive HF (hs-CRP: 3.47 vs. 2.29 mg/L, *p* < 0.001; D-dimer: 0.41 vs. 0.32 mg/L FEU, *p* = 0.001; hs-IL-6: 3.41 vs. 2.37 pg/mL, *p* < 0.001; Fig. 3 [hs-CRP only] and Supplementary Table S4 ).

High-CHADS2 scores were associated with significantly higher baseline levels of D-dimer and hs-IL-6 than low CHADS2 scores (D-dimer: 0.31, 0.32, and 0.36 mg/L FEU for low, moderate, and high-CHADS2 scores, respectively, *p* < 0.001; hs-IL-6: 2.27, 2.48, and 2.73 pg/mL for low, moderate, and high-CHADS2 scores, respectively, *p* < 0.001; Supplementary Table S5 ).

**Fig. 4:**
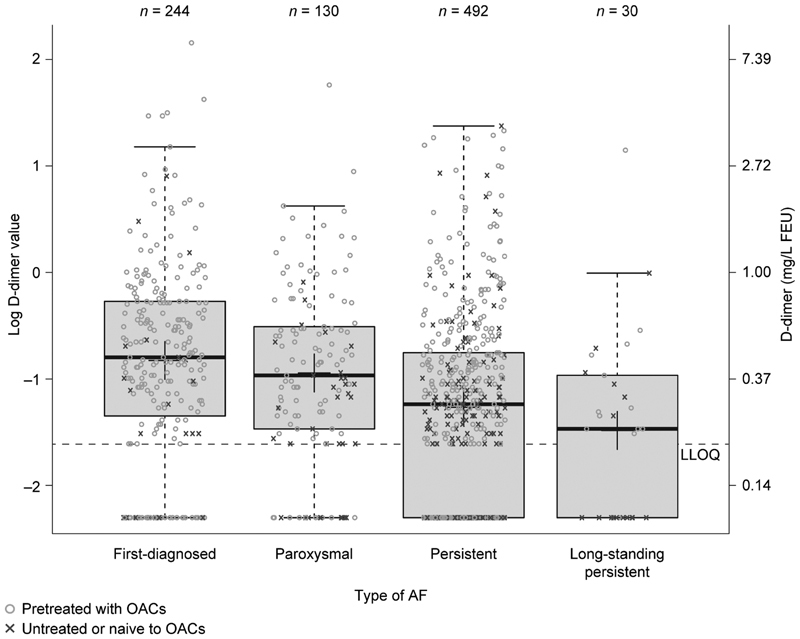
Influence of type of AF on levels of D-dimer at baseline. AF, atrial fibrillation; FEU, fibrinogen equivalent units; LLOQ, lower limit of quantification; OAC, oral anticoagulant. Note: Upper lines of the box denote the upper quartiles, mid-lines denote the medians and lower lines denote the lower quartiles; crosses denote the geometric means, and upper and lower lines denote maximum and minimum values, excluding outliers (i.e., values that are >1.5 times the interquartile range further apart from the box).

There was a significant negative relationship between baseline levels of D-dimer and increasing persistence of AF (0.43, 0.39, 0.28, and 0.24 mg/L FEU for first-diagnosed, paroxysmal, persistent, and long-standing persistent AF, respectively, *p* < 0.001; Supplementary Table S6 ). A similar negative relationship was also found between the persistence of AF and levels of hs-IL-6, although baseline levels of hs-IL-6 were highest in patients with paroxysmal AF (2.71, 2.96, 2.36, and 2.03 pg/mL for first-diagnosed, paroxysmal, persistent, and long-standing persistent AF, respectively, *p* < 0.001; Fig. 4 [D-dimer only] and Supplementary Table S6 ).

### Relationship between Biomarker Levels at Baseline and Clinical Events during the Study

#### Bleeding Events

The AIC-based analysis revealed hs-CRP baseline levels together with the presence of a prior stroke and a prior TIA as the best set of potential predictors for bleeding events. However, none of these variables was significantly related to the occurrence of bleeding events ( Table 2 ), indicating no prognostic value of the biomarkers or of any of the clinical or demographic covariates.

**Table 2:** Results of multivariate models for bleeding events, cardioversion success and time to cardioversion by biomarker baseline levels, and demographic and clinical covariates

Event	*n*	Parameter a	Effect	OR	95% CI	*p-* Value
Logistic models						
Bleeding event (yes/no)	828 (96 cases)	Log hs-CRP	0.12	1.13	0.96–1.33	0.153
		Prior stroke = yes	0.74	2.10	0.80–5.54	0.134
		Prior TIA = yes	0.71	2.03	0.78–5.32	0.149
Cardioversion success (yes/no)	693 (594 cases)	History of diabetes mellitus = yes	−0.54	0.58	0.34–0.98	0.042
		Treatment = rivaroxaban	0.59	1.81	1.16–2.82	0.009
		Race = Asian	−1.08	0.34	0.19–0.63	0.001
		Race = Black	−1.28	0.28	0.07–1.12	0.072
		Race = Other	−0.96	0.38	0.13–1.13	0.082
**Endpoint**	***n***	** Parameter a**	**Effect**	**95% CI**		***p-*** **Value**
Linear model						
Log time to first cardioversion under treatment	693	Log hs-CRP	0.05	0.02–0.09		0.004
		Log hs-IL-6	−0.12	−0.18 to −0.06		<0.001
		Log F1.2	0.09	0.05–0.13		<0.001
		Log TAT	−0.06	−0.10 to −0.02		0.003
		History of arterial hypertension = yes	−0.05	−0.13 to 0.02		0.167
		History of diabetes mellitus = yes	−0.08	−0.17 to 0.02		0.101
		Prior myocardial infarction = yes	0.18	0.04–0.32		0.012
		Treatment = rivaroxaban	−0.17	−0.24 to −0.10		<0.001
		Cardioversion strategy = Delayed	2.49	2.42–2.56		<0.001

Abbreviations: CI, confidence interval; F1.2, prothrombin fragment 1 + 2; HF, heart failure; hs-CRP, high-sensitivity C-reactive protein; hs-IL-6, high-sensitivity interleukin-6; OR, odds ratio; TAT, thrombin–anti-thrombin III complex; TIA, transient ischemic attack.

a Parameter combination chosen by stepwise AIC (Akaike's information criterion) forward selection.

#### Cardioversion Success

The analysis of the success of the cardioversion did not reveal any additional benefit of biomarkers. The AIC-optimal model contained the history of diabetes mellitus (decreased chance for a successful cardioversion, odds ratio [OR] = 0.58, *p* = 0.042), treatment (increased chance if treated with rivaroxaban, OR = 1.81, *p* = 0.009), and race (decreased chance for Asian patients in comparison with White patients, OR = 0.34, *p* = 0.001) as significant predictors ( Table 2 ).

#### Time to First Cardioversion under Treatment

The AIC-optimal model for the log-transformed time to cardioversion contained all biomarkers with the exception of D-dimer, in addition to the cardioversion strategy, treatment, history of arterial hypertension, history of diabetes mellitus, and prior MI ( Table 2 ). Even though all of the contained biomarkers were significantly related to the time to cardioversion, the amount of explained variability could only be increased by 1.1% points in comparison with the model that contained only the cardioversion strategy as a predictor (88.1 vs. 87.0%), which indicates no relevant additional value of the biomarkers.

Additional descriptive statistics for the biomarker baseline levels in relation to the clinical events described in this section are provided in Supplementary Table S7 .

### Influence of Treatment with Rivaroxaban or a VKA on Biomarker Levels

**Fig. 5:**
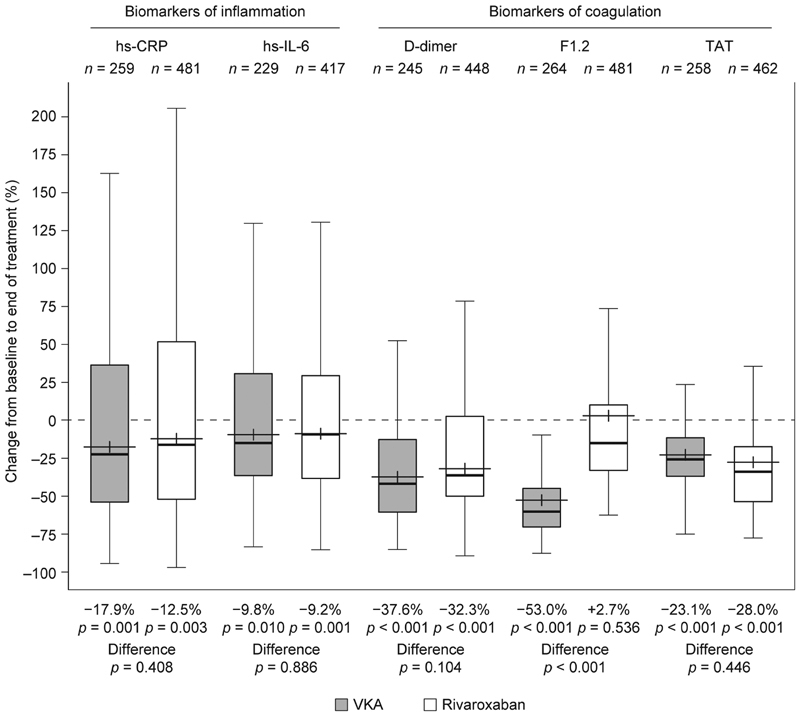
Relative changes of biomarker levels from baseline to the end of treatment, model-adjusted for biomarker baseline level, and treatment duration. F1.2, prothrombin fragment 1 + 2; hs-CRP, high-sensitivity C-reactive protein; hs-IL-6, high-sensitivity interleukin-6; TAT, thrombin–anti-thrombin III complex; VKA, vitamin-K antagonist. Note: Upper lines of the box denote the upper quartiles, mid-lines denote the medians and lower lines denote the lower quartiles; crosses denote the geometric means, and upper and lower lines denote maximum and minimum values, excluding outliers (i.e., values that are >1.5 times the interquartile range further apart from the box).

Levels of D-dimer, TAT, hs-CRP, and hs-IL-6 decreased from baseline to the end of treatment in both the rivaroxaban group and in the VKA group, with the greatest reductions of 32.3% in the rivaroxaban group and 37.7% in the VKA group being observed for D-dimer ( Fig. 5 ; Supplementary Table S8 ). After adjusting for biomarker baseline levels and treatment duration, there was no significant difference between treatment groups in the reductions observed for these biomarkers. F1.2 levels slightly increased in the rivaroxaban group from baseline to end of treatment, which was significantly different from the VKA treatment arm, in which F1.2 levels decreased (rivaroxaban: 2.7% [95% confidence interval (CI): −5.6 to 11.7%]; VKA: −53% [95% CI: −58.1 to −47.3%]; *p* < 0.001).

### Factors Influencing Changes in Biomarker Levels from Baseline to the End of Treatment

#### Pretreatment with OACs

After adjusting for baseline levels of each biomarker and the duration of treatment, there was a significant association between pretreatment with OACs and changes from baseline to the end of treatment in the levels of D-dimer ( *p* < 0.001, Table 3 ) and F1.2 ( *p* = 0.002, Table 3 ). No significant association was found between pretreatment with OACs and changes from baseline to the end of treatment in levels of hs-CRP, hs-IL-6, or TAT. The impact of pretreatment with OACs on the changes from baseline to the end of treatment in biomarker levels did not differ significantly with regard to treatment with rivaroxaban or VKA. As summarized in Table 3 , in the rivaroxaban group, D-dimer levels decreased from baseline by 36.0% in patients who were untreated or naive to OACs and by 23.1% in patients who had received pretreatment with OACs. In the VKA group, D-dimer levels decreased from baseline by 42.6% (patients untreated or naive to OACs) and by 23.4% (patients pretreated with OACs). In the rivaroxaban group, F1.2 levels remained nearly unchanged (patients untreated or naive to OACs) or increased by 15% (patients pretreated with OACs). In the VKA group, F1.2 levels decreased by 58.7% (patients untreated or naive to OACs) and by 36.8% (patients pretreated with OACs). With the exception of F1.2, there were no significant differences between the treatment arms ( Table 3 ).

**Table 3:** Association of biomarker changes from baseline to end of treatment with clinical variables (adjusted for biomarker baseline level and treatment duration)

Biomarker	Factor	*p* -Value a	Subgroup	Rivaroxaban	VKA	*t* -Statistic	*p* -Value b
hs-CRP	OAC taken prior to study	0.085	OAC taken prior to study = no	−14.5% (95% CI: −23.0 to − 5.1%; *n* = 348)	−23.6% (95% CI: −33.9 to −11.8%; *n* = 180)	1.242	0.215
			OAC taken prior to study = yes	−7.4% (95% CI: −21.7 to +9.5%; *n* = 133)	−2.7% (95% CI: −21.8 to 21.1%; *n* = 79)	−0.350	0.726
	History of congestive HF	0.721	History of congestive HF = no	−14.8% (95% CI: −22.7 to −6.1%; *n* = 399)	−15.3% (95% CI: −25.6 to −3.6%; *n* = 225)	0.077	0.938
			History of congestive HF = yes	−0.7% (95% CI: −19.9 to +23.1%; *n* = 82)	−32.9% (95% CI: −51.8 to −6.5%; *n* = 34)	1.950	0.052
	Type of AF	0.786	Type of AF = first diagnosed	−5.5% (95% CI: −19.6 to +11.1%; *n* = 143)	−37.2% (95% CI: −51.0 to −19.6%; *n* = 61)	2.715	0.007
			Type of AF = paroxysmal	−11.5% (95% CI: −30.8 to +13.2%; *n* = 62)	−1.0% (95% CI: −25.6% to 31.6; *n* = 46)	−0.583	0.560
			Type of AF = persistent	−15.3% (95% CI: −24.9 to −4.6%; *n* = 264)	−12.2% (95% CI: −25.7 to 3.6%; *n* = 137)	−0.343	0.731
			Type of AF= long-standing persistent	−26.3% (95% CI: −58.9 to +32.0%; *n* = 11)	−29.6% (95% CI: −60.7 to 26.1%; *n* = 11)	0.108	0.914
D-dimer	OAC taken prior to study	< 0.001	OAC taken prior to study = no	−36.0% (95% CI: −40.3 to −31.3%; *n* = 317)	−42.6% (95% CI: −47.8 to −37.0%; *n* = 171)	1.832	0.067
			OAC taken prior to study = yes	−23.1% (95% CI: −31.1 to −14.2%; *n* = 131)	−23.4% (95% CI: −33.8 to −11.4%; *n* = 74)	0.045	0.964
	History of congestive HF	0.117	History of congestive HF = no	−33.9% (95% CI: −38.1 to −29.5%; *n* = 376)	−37.7% (95% CI: −42.8 to −32.2%; *n* = 216)	1.079	0.281
			History of congestive HF = yes	−23.1% (95% CI: −33.6 to −10.8%; *n* = 72)	−37.4% (95% CI: −50.4 to −21.1%; *n* = 29)	1.486	0.138
	Type of AF	0.979	Type of AF = first-diagnosed	−33.2% (95% CI: −40.2 to −25.3%; *n* = 131)	−34.4% (95% CI: −44.5 to −22.3%; *n* = 56)	0.177	0.860
			Type of AF = paroxysmal	−32.1% (95% CI: −42.6 to −19.7%; *n* = 56)	−37.7% (95% CI: −48.6 to −24.7%; *n* = 43)	0.674	0.501
			Type of AF = persistent	−32.1% (95% CI: −37.3 to −26.5%; *n* = 251)	−39.1% (95% CI: −45.5 to −32.0%; *n* = 131)	1.574	0.116
			Type of AF = long-standing persistent	−27.3% (95% CI: −52.1 to +10.4%; *n* = 9)	−32.7% (95% CI: −53.9 to −1.8%; *n* = 11)	0.272	0.785
F1.2	OAC taken prior to study	0.002	OAC taken prior to study = no	−1.8% (95% CI: −11.2 to +8.5%; *n* = 347)	−58.7% (95% CI: −63.9 to −52.6%; *n* = 183)	10.115	<0.001
			OAC taken prior to study = yes	+15.0% (95% CI: −2.4 to +35.4%; *n* = 134)	−36.8% (95% CI: −48.8 to −22.1%; *n* = 81)	4.560	<0.001
	History of congestive HF	0.033	History of congestive HF = no	+1.4% (95% CI: −7.5 to +11.1%; *n* = 400)	−55.8% (95% CI: −60.9 to −50.1%; *n* = 231)	10.687	<0.001
			History of congestive HF = yes	+9.6% (95% CI: −10.6 to +34.3%; *n* = 81)	−27.6% (95% CI: −47.3 to −0.3%; *n* = 33)	2.145	0.032
	Type of AF	0.754	Type of AF = first-diagnosed	+11.7% (95% CI: −4.4 to +30.5%; *n* = 142)	−53.3% (95% CI: −63.1 to −40.7%; *n* = 61)	6.047	<0.001
			Type of AF = paroxysmal	+0.6% (95% CI: −20.4 to +27.3%; *n* = 62)	−47.0% (95% CI: −59.8 to −30.3%; *n* = 45)	3.490	0.001
			Type of AF = persistent	−0.5% (95% CI: −11.3 to +11.5%; *n* = 265)	−54.5% (95% CI: −61.1 to −46.9%; *n* = 142)	7.917	<0.001
			Type of AF = long-standing persistent	−20.8% (95% CI: −54.6 to +38.2%; *n* = 11)	−46.3% (95% CI: −68.5 to −8.4%; *n* = 12)	0.989	0.323

Abbreviations: AF, atrial fibrillation; ANCOVA, analysis of covariance; CI, confidence interval; F1.2, prothrombin fragment 1 + 2; HF, heart failure; hs-CRP, high-sensitivity C-reactive protein; hs-IL-6, high-sensitivity interleukin-6; OAC: oral anticoagulant; TAT, thrombin–anti-thrombin III complex; VKA, vitamin-K antagonist.

Note: Results for further clinical variables and results for the biomarkers hs-IL-6 and TAT are provided in the supplementary material.

a *p* -Values were calculated from an ANCOVA that contained the biomarker baseline level, treatment duration and the treatment arm in addition to the respective factor variable.

b *p* -Values and *t* -statistics were calculated from a linear model with the biomarker baseline level, treatment duration, treatment arm and the respective subgroup variable (as a main effect and in interaction with treatment) as independent variables. Consequently, the given mean changes are adjusted for treatment duration and biomarker baseline level.

#### History of Congestive HF

After adjusting for baseline levels of each biomarker and treatment duration, in the rivaroxaban group, there was a significant association between a history of congestive HF and changes in levels of F1.2 from baseline to the end of treatment ( *p* = 0.033, Table 3 ). In the rivaroxaban group, F1.2 levels were increased by 9.6% in patients with a history of congestive HF, and by 1.4% in those with no history of congestive HF. In the VKA group, F1.2 levels decreased from baseline by 27.6% in those with a history of congestive HF and by 55.8% in patients with no history of congestive HF ( Table 3 ).

#### Type of AF

After adjusting for baseline levels and treatment duration, there was a significant difference between the treatment arms in hs-CRP changes in patients with a first-diagnosed AF (rivaroxaban: −5.5% [95% CI: −19.6% to 11.1%; VKA: −37.2% [95% CI: −51.0 to −19.6%; *p* = 0.007; Table 3 ).

No significant relationship between the changes in biomarker levels and the cardioversion success or the cardioversion strategy was found after adjusting for differences in baseline levels and treatment duration ( Supplementary Table S9 ).

## Discussion

The X-VeRT post hoc analyses have demonstrated that, in patients with AF undergoing planned cardioversion, anticoagulation with rivaroxaban is associated with a reduction in levels of key biomarkers of both coagulation (D-dimer and TAT) and inflammation (hs-IL-6 and hs-CRP). The results were similar to those observed in the VKA group. In addition, F1.2 levels were lower in VKA-treated patients than rivaroxaban-treated patients. These results, therefore, confirm the expected pharmacodynamic effects of rivaroxaban in this patient population.

The observed reductions in biomarkers of both coagulation and inflammation highlight the important bidirectional link between coagulation and inflammation and support other data demonstrating the potential of rivaroxaban and possibly of VKAs to have anti-inflammatory effects in addition to their anticoagulant actions. 22 23 24 25 26 The decrease of the mentioned biomarkers during rivaroxaban treatment has also been shown in a similar study in AF. 16 Here, von Willebrand's factor (vWF) and plasminogen activator inhibitor-1 were measured at baseline and after 6 weeks of treatment in addition to the biomarkers described here. The hs-CRP, D-dimer, vWF, and TAT showed a significant decrease in the mean levels from baseline to end of treatment with rivaroxaban. Although it is already well established that inflammation leads to activation of the coagulation cascade, 27 28 29 there has been recent focus on the coagulation cascade triggering the activation of inflammatory pathways. Thrombin, factor Xa, and fibrin all stimulate the production of the proinflammatory cytokines IL-6 and IL-8, 22 27 30 and treatment with enoxaparin has been associated with a reduction in hs-CRP levels from baseline in patients with AF who underwent cardioversion. 13

In addition to data indicating a role for inflammation in the pathogenesis of AF, 8 9 10 11 there is evidence suggesting that inflammation can be a consequence of AF. 31 32 33 It is, therefore, possible that the reduction in inflammatory biomarkers observed in this substudy was, in part, a consequence of the restoration of sinus rhythm after cardioversion. In a small study of patients with persistent AF, hs-CRP levels in patients who remained in sinus rhythm after cardioversion gradually decreased and were significantly lower at the final evaluation than at baseline before cardioversion; hs-CRP levels did not change significantly in patients with AF recurrence. 32 These data are not supported, however, by a larger study on a range of inflammatory biomarkers before and after cardioversion. 34 Overall, it is unlikely that an effect of successful cardioversion accounts for the entirety of the reductions observed in this study.

AF is associated with a prothrombotic state, 6 and induces venous stasis and elevated venous pressure, which is thought to subsequently lead to abnormal changes in blood constituents, leading to hemostatic and platelet activation, as well as inflammation, and growth factor changes. 8 However, it has previously been demonstrated that levels of D-dimer remain elevated even after successful cardioversion. 35 Furthermore, rivaroxaban treatment has been associated with reductions in D-dimer, TAT, and F1.2 levels in treatment-naive patients with AF, 36 37 suggesting that it is possible that at least some of the effects of rivaroxaban on these biomarkers in the current study are mediated by anticoagulant effects.

The effects of pretreatment with OACs on baseline levels of D-dimer and F1.2 are in agreement with other observations and, given the mechanism of action of these drugs, are as expected. 38 The mean reduction in levels of D-dimer and F1.2 in response to treatment with rivaroxaban or a VKA was significantly smaller in patients who had received pretreatment with OACs than in patients who were untreated or naive to OACs. Pretreatment with OACs, therefore, needs to be considered when measuring selected coagulation biomarkers at baseline.

Treatment with a VKA, but not with rivaroxaban, was associated with a reduction from baseline in levels of F1.2 in the present study. It appears that this biomarker might be differently sensitive to both treatment regimens. One hypothesis is that this may be related to the different pharmacokinetic profiles of rivaroxaban and VKAs in terms of interaction with the kinetics of F1.2, which is a marker of thrombin activation. For example, the mean half-life of rivaroxaban is approximately 5 to 13 hours, whereas the mean half-life of warfarin is approximately 40 hours. 39 40 It has been hypothesized that different effects of rivaroxaban and warfarin on F1.2 levels and, therefore, on thrombin generation at steady-state might be associated with differences in the safety profiles of the two drugs. 41 For example, compared with warfarin, rivaroxaban, and other NOACs are associated with lower rates of intracerebral hemorrhage (ICH) and better outcomes (e.g., hematoma expansion) after ICH. 42 43 44 The lower F1.2 levels in patients receiving warfarin versus rivaroxaban could provide a possible explanation for this clinical difference. Further studies, ideally relating ICH outcomes to F1.2 levels, are needed to test this.

There is increasing evidence that inflammatory mediators play an important role in the development of congestive HF. 45 In patients with HF, the immune system is chronically activated, with increased circulating levels of the proinflammatory cytokines tumor necrosis factor, IL-1 and IL-6 in both ischemic and nonischemic HF. 46 Elevated levels of CRP have also been associated with high mortality and high rates of readmission in patients with HF. 47 The elevated baseline levels of the inflammatory biomarkers hs-CRP and hs-IL-6 observed in patients with a history of congestive HF in this study are consistent with an underlying inflammatory state in this group of patients. Given that F1.2 is a known biomarker of coagulation, it was expected that patients with a history of congestive HF would have higher F1.2 levels at baseline than those without a history of congestive HF, but this was not observed.

The observation that patients with newly diagnosed AF had higher D-dimer levels at baseline than patients with long-standing AF was unexpected because long-standing AF is thought to be associated with a prothrombotic state and higher levels of D-dimer than acute AF. 48 However, recent large observational studies have demonstrated that acute-onset AF is associated with a higher risk of stroke than long-standing AF, 49 and elevated levels of D-dimer have also been associated with an increased risk of stroke in patients with AF. 50 51 Our findings are consistent with these observations and demonstrate that disease effects need to be considered when evaluating biomarkers. In addition, the higher D-dimer levels might be related to less prior use of OACs in patients with newly diagnosed AF than in patients with long-standing AF.

Also unexpected were the decreased hs-IL-6 and F1.2 levels in patients with long-standing AF. It has been described that long duration of AF is associated with high–hs-CRP levels and large–left atrial dimensions, supporting a link between the burden of AF, inflammation, and structural remodeling. 52 IL-6 physiologically stimulates the synthesis of several acute-phase reaction proteins, such as CRP, so that one would also expect increased IL-6 levels in long-standing AF. However, the findings in this study show the opposite effect regarding IL-6 and no relevant effect on hs-CRP. The pretreatment with OACs did not significantly influence baseline levels of hs-IL-6, so this cannot explain these findings regarding hs-IL-6; however, F1.2 and D-dimer levels seem to be affected by prior OAC intake. Further studies need to be done to explain or to confirm these findings.

None of the biomarkers showed any prognostic or predictive value with regard to bleeding events or the probability for cardioversion success. Baseline levels of all biomarkers except D-dimer were significantly associated with time to cardioversion. However, effect sizes were small, and no discriminatory power of the biomarkers was detected. In the X-TRA biomarker substudy, high levels of inflammatory biomarkers in patients with AF who had left atrial/left atrial appendage thrombus were associated with the thrombus being completely resolved or reduced with rivaroxaban treatment and, therefore, also showed potential as predictive biomarkers. 16 These correlates require additional investigation to assess their clinical utility.

A key strength of the present analyses was the large sample size. In some cases, even small changes in biomarker levels were found to be statistically significant. However, it has to be noted that, even though a large number of analyses were performed, no formal adjustment for multiple testing was performed owing to the exploratory nature of the analyses. Thus, when interpreting the results, the potential for false-positive results due to multiplicity must be considered. Moreover, owing to the post hoc nature of the analyses and the fact that biomarkers were only collected from a subset of the total study population, it has to be noted that balance between the two treatment groups with regard to demographics, clinical variables, and biomarker baseline levels was not ensured (summary of baseline characteristics: Supplementary Table S2 ). All analyses of biomarker changes from baseline to end of treatment were adjusted for biomarker levels at baseline, making it unlikely that a regression to the mean effect explains the reduction in biomarker levels that was observed from baseline to the end of treatment.

## Conclusion

In conclusion, treatment with rivaroxaban or a VKA resulted in a significant reduction from baseline in levels of biomarkers of coagulation (D-dimer and TAT) and inflammation (hs-IL-6 and hs-CRP), with no significant difference between treatment groups. F1.2 levels were reduced from baseline in patients randomized to receive a VKA but not in those randomized to receive rivaroxaban, providing hypothesis-generating insights into the possible mechanisms driving the safety differences between VKAs and rivaroxaban. The results presented here also highlight the need to consider pretreatment with OACs and clinical factors (e.g., type of AF and history of congestive HF) when evaluating biomarkers of coagulation and inflammation, and invite further investigation to confirm the potential prognostic or predictive value of these biomarkers in patients with AF.
