# Supplementary material for: Effects of Rivaroxaban on Biomarkers of Coagulation and Inflammation: A Post Hoc Analysis of the X-VeRT Trial
Source: TH Open. 2020 Jan 23;4(1):e20–32. doi: 10.1055/s-0040-1701206 (PMC6978177; doi:10.1055/s-0040-1701206)
Supplement: Supplementary file 1 — Supplementary Material [file 10-1055-s-0040-1701206-s190054.pdf]

Supplementary Table S1 Number of biomarker measurements by time point and treatment arm

Biomarker	Baseline (VKA/rivaroxaban)	End of treatment (VKA/rivaroxaban)
D-dimer	901 (299/602)	882 (285/597)
F1.2	950 (319/631)	909 (292/617)
hs-CRP	950 (318/632)	905 (287/618)
hs-IL-6	894 (298/596)	837 (268/569)
TAT	931 (311/620)	895 (293/602)
Total number of subjects	958 (320/638)	918 (294/624)

Abbreviations: F1.2, prothrombin fragment 1+2; hs-CRP, high-sensitivity C-reactive protein; hs-IL-6, high-sensitivity interleukin-6; TAT, thrombin–anti-thrombin III complex; VKA, vitamin K antagonist.

Supplementary Table S2 Baseline characteristics per treatment arm

Baseline characteristic	Level	VKA $n = 320$ (%)	Rivaroxaban $n = 638$ (%)	*p*-Value[a] (difference between treatment arms)
Sex	Male	233 (72.8)	462 (72.4)	0.939
	Female	87 (27.2)	176 (27.6)	
Age (y)	18–< 40	7 (2.2)	13 (2.0)	0.301
	40–< 65	151 (47.2)	261 (40.9)	
	65–< 75	102 (31.9)	233 (36.5)	
	≥ 75	60 (18.8)	131 (20.5)	
Race	White	277 (86.6)	560 (87.8)	0.757
	Asian	30 (9.4)	48 (7.5)	
	Black	5 (1.6)	10 (1.6)	
	Other	8 (2.5)	20 (3.1)	
BMI (kg/m^2)	< 25	55 (17.2)	117 (18.3)	0.762
	25–< 35	204 (63.8)	411 (64.4)	
	≥ 35	60 (18.8)	109 (17.1)	
Creatinine clearance (mL/min)	< 30	1 (0.3)	0 (0.0)	0.167
	30–< 50	21 (6.6)	44 (6.9)	
	50–< 80	115 (35.9)	196 (30.7)	
	≥ 80	181 (56.6)	394 (61.8)	
Prior stroke	No	309 (96.6)	614 (96.2)	0.857
	Yes	11 (3.4)	24 (3.8)	
Prior TIA	No	306 (95.6)	621 (97.3)	0.177
	Yes	14 (4.4)	17 (2.7)	
Prior non-CNS systemic embolism	No	315 (98.4)	631 (98.9)	0.548
	Yes	5 (1.6)	7 (1.1)	
History of congestive HF	No	278 (86.9)	526 (82.4)	0.093
	Yes	42 (13.1)	112 (17.6)	
History of arterial hypertension	No	95 (29.7)	229 (35.9)	0.060
	Yes	225 (70.3)	409 (64.1)	
History of diabetes mellitus	No	258 (80.6)	517 (81.0)	0.931
	Yes	62 (19.4)	121 (19.0)	
Prior vascular disease	No	283 (88.4)	559 (87.6)	0.754
	Yes	37 (11.6)	79 (12.4)	

Supplementary Table S2 (*Continued*)

Baseline characteristic	Level	VKA<br>$n = 320$ (%)	Rivaroxaban<br>$n = 638$ (%)	*p*-Value[a] (difference between treatment arms)
Prior MI	No	298 (93.1)	590 (92.5)	0.793
	Yes	22 (6.9)	48 (7.5)	
Type of AF	First diagnosed	71 (22.2)	190 (29.8)	0.001
	Paroxysmal	56 (17.5)	84 (13.2)	
	Persistent	170 (53.1)	348 (54.5)	
	Long-standing persistent	19 (5.9)	15 (2.4)	
	Missing	4 (1.2)	1 (0.2)	
CHADS2 score	Low	67 (20.9)	162 (25.4)	0.285
	Moderate	130 (40.6)	237 (37.1)	
	High	123 (38.4)	239 (37.5)	
OAC taken prior to study	No	218 (68.1)	468 (73.4)	0.095
	Yes	102 (31.9)	170 (26.6)	
Cardioversion strategy	Early	151 (47.2)	299 (46.9)	0.945
	Delayed	169 (52.8)	339 (53.1)	
Cardioversion success	No	31 (9.7)	70 (11.0)	0.219
	Yes	153 (47.8)	466 (73.0)	
**Biomarker**	**Statistic**	**VKA<br>($n = 320$)**	**Rivaroxaban<br>($n = 638$)**	***p*-Value[b] (difference between treatment arms)**
hs-CRP (mg/L)	Geometric mean	2.25	2.38	0.519
	95% CI	1.95–2.60	2.15–2.64	
	n	318	632	
D-dimer (mg/L FEU)	Geometric mean	0.31	0.34	0.099
	95% CI	0.28–0.34	0.32–0.37	
	n	299	602	
hs-IL-6 (pg/mL)	Geometric mean	2.49	2.53	0.725
	95% CI	2.28–2.71	2.38–2.69	
	n	298	596	
F1.2 (pmol/L)	Geometric mean	164.46	162.73	0.886
	95% CI	145.53–185.85	149.94–176.61	
	n	319	631	
TAT (µg/L)	Geometric mean	3.87	4.21	0.279
	95% CI	3.45–4.34	3.84–4.60	
	n	311	620	

Abbreviations: AF, atrial fibrillation; BMI, body mass index; CI, confidence interval; CHADS2, congestive HF, hypertension, age ≥ 75 years, diabetes mellitus, prior stroke, thromboembolism or TIA; CNS, central nervous system; F1.2, prothrombin fragment 1 + 2; FEU, fibrinogen equivalent units; HF, heart failure; hs-CRP, high-sensitivity C-reactive protein; hs-IL-6, high-sensitivity interleukin-6; MI, myocardial infarction; OAC, oral anticoagulant; TAT, thrombin–anti-thrombin III complex; TIA, transient ischemic attack; VKA, vitamin K antagonist.

[a]Calculated from Fisher's exact test.

[b]Calculated from a *t*-test on the log-transformed biomarker levels.

Supplementary Table S3 Mean biomarker levels at baseline by prior OAC intake adjusted for demographic covariates

Biomarker	No	Yes	*p*-Value[a]
hs-CRP (mg/L)	2.51 (95% CI: 2.26–2.78; *n* = 679)	2.27 (95% CI: 1.93–2.66; *n* = 271)	0.270
D-dimer (mg/L FEU)	0.40 (95% CI: 0.38–0.43; *n* = 638)	0.20 (95% CI: 0.19–0.22; *n* = 263)	<0.001
hs-IL-6 (pg/mL)	2.56 (95% CI: 2.42–2.71; *n* = 637)	2.41 (95% CI: 2.21–2.64; *n* = 257)	0.278
F1.2 (pmol/L)	214.99 (95% CI: 198.41–232.97; *n* = 679)	87.23 (95% CI: 77.16–98.62; *n* = 271)	<0.001
TAT (μg/L)	5.71 (95% CI: 4.70–6.93; *n* = 667)	4.48 (95% CI: 3.60–5.57; *n* = 264)	0.003

Abbreviations: CI, confidence interval; F1.2, prothrombin fragment 1 + 2; FEU, fibrinogen equivalent units; hs-CRP, high-sensitivity C-reactive protein; hs-IL-6, high-sensitivity interleukin-6; OAC, oral anticoagulant; TAT, thrombin–anti-thrombin III complex.
[a]Adjusted for demographic covariates as given in ►**Table 1** by a linear model.

Supplementary Table S4 Mean biomarker levels at baseline by medical history adjusted for demographic covariates

Biomarker	Medical history	No	Yes	*p*-Value[a]
hs-CRP (mg/L)	Prior stroke	2.39 (95% CI: 2.18–2.62; *n* = 916)	3.96 (95% CI: 2.58–6.08; *n* = 34)	0.024
	Prior TIA	2.41 (95% CI: 2.20–2.64; *n* = 920)	3.44 (95% CI: 2.17–5.46; *n* = 30)	0.136
	Prior non-CNS systemic embolism	2.44 (95% CI: 2.23–2.68; *n* = 938)	2.04 (95% CI: 0.99–4.19; *n* = 12)	0.621
	History of congestive HF	2.29 (95% CI: 2.08–2.52; *n* = 796)	3.47 (95% CI: 2.82–4.27; *n* = 154)	<0.001
	History of arterial hypertension	2.45 (95% CI: 2.10–2.85; *n* = 321)	2.43 (95% CI: 2.18–2.72; *n* = 629)	0.960
	History of diabetes mellitus	2.46 (95% CI: 2.23–2.72; *n* = 768)	2.35 (95% CI: 1.93–2.86; *n* = 182)	0.669
	Prior vascular disease	2.39 (95% CI: 2.18–2.63; *n* = 837)	2.88 (95% CI: 2.25–3.69; *n* = 113)	0.155
	Prior MI	2.39 (95% CI: 2.18–2.63; *n* = 882)	3.19 (95% CI: 2.33–4.36; *n* = 68)	0.078
D-dimer (mg/L FEU)	Prior stroke	0.33 (95% CI: 0.31–0.35; *n* = 869)	0.43 (95% CI: 0.32–0.57; *n* = 32)	0.097
	Prior TIA	0.33 (95% CI: 0.31–0.35; *n* = 871)	0.46 (95% CI: 0.33–0.62; *n* = 30)	0.042
	Prior non-CNS systemic embolism	0.33 (95% CI: 0.31–0.35; *n* = 890)	0.34 (95% CI: 0.21–0.57; *n* = 11)	0.895
	History of congestive HF	0.32 (95% CI: 0.30–0.34; *n* = 760)	0.41 (95% CI: 0.36–0.47; *n* = 141)	0.001
	History of arterial hypertension	0.34 (95% CI: 0.31–0.38; *n* = 302)	0.33 (95% CI: 0.30–0.35; *n* = 599)	0.365
	History of diabetes mellitus	0.34 (95% CI: 0.32–0.36; *n* = 729)	0.31 (95% CI: 0.27–0.35; *n* = 172)	0.250
	Prior vascular disease	0.33 (95% CI: 0.31–0.35; *n* = 793)	0.36 (95% CI: 0.30–0.42; *n* = 108)	0.379
	Prior MI	0.33 (95% CI: 0.31–0.35; *n* = 837)	0.39 (95% CI: 0.31–0.48; *n* = 64)	0.126
hs-IL-6 (pg/mL)	Prior stroke	2.49 (95% CI: 2.37–2.61; *n* = 862)	3.38 (95% CI: 2.62–4.35; *n* = 32)	0.020
	Prior TIA	2.51 (95% CI: 2.39–2.64; *n* = 866)	2.61 (95% CI: 1.99–3.43; *n* = 28)	0.787
	Prior non-CNS systemic embolism	2.51 (95% CI: 2.40–2.64; *n* = 883)	2.62 (95% CI: 1.70–4.04; *n* = 11)	0.851
	History of congestive HF	2.37 (95% CI: 2.25–2.50; *n* = 750)	3.41 (95% CI: 3.03–3.84; *n* = 144)	<0 0.001
	History of arterial hypertension	2.45 (95% CI: 2.25–2.67; *n* = 301)	2.55 (95% CI: 2.40–2.71; *n* = 593)	0.494
	History of diabetes mellitus	2.52 (95% CI: 2.39–2.66; *n* = 719)	2.50 (95% CI: 2.24–2.80; *n* = 175)	0.929
	Prior vascular disease	2.49 (95% CI: 2.36–2.62; *n* = 785)	2.74 (95% CI: 2.39–3.16; *n* = 109)	0.197
	Prior MI	2.49 (95% CI: 2.37–2.62; *n* = 829)	2.82 (95% CI: 2.36–3.38; *n* = 65)	0.192

Supplementary Table S4 (*Continued*)

Biomarker	Medical history	No	Yes	*p*-Value[a]
F1.2 (pmol/L)	Prior stroke	166.38 (95% CI: 153.95–179.81; $n = 915$)	198.89 (95% CI: 139.49–283.58; $n = 35$)	0.332
	Prior TIA	165.41 (95% CI: 153.14–178.66; $n = 919$)	252.72 (95% CI: 172.84–369.51; $n = 31$)	0.030
	Prior non-CNS systemic embolism	167.30 (95% CI: 154.93–180.65; $n = 938$)	188.08 (95% CI: 102.77–344.20; $n = 12$)	0.705
	History of congestive HF	172.07 (95% CI: 158.66–186.62; $n = 798$)	144.13 (95% CI: 121.01–171.66; $n = 152$)	0.061
	History of arterial hypertension	175.43 (95% CI: 154.78–198.83; $n = 321$)	163.85 (95% CI: 149.70–179.33; $n = 629$)	0.364
	History of diabetes mellitus	168.73 (95% CI: 155.17–183.47; $n = 770$)	162.84 (95% CI: 138.89–190.92; $n = 180$)	0.689
	Prior vascular disease	168.30 (95% CI: 155.41–182.25; $n = 835$)	161.37 (95% CI: 131.50–198.02; $n = 115$)	0.698
	Prior MI	167.88 (95% CI: 155.25–181.54; $n = 881$)	162.76 (95% CI: 125.59–210.93; $n = 69$)	0.818
TAT (µg/L)	Prior stroke	5.23 (95% CI: 4.32–6.33; $n = 900$)	6.27 (95% CI: 4.15–9.47; $n = 31$)	0.374
	Prior TIA	5.29 (95% CI: 4.38–6.39; $n = 901$)	5.63 (95% CI: 3.66–8.67; $n = 30$)	0.764
	Prior non-CNS systemic embolism	5.27 (95% CI: 4.37–6.37; $n = 919$)	6.88 (95% CI: 3.61–13.11; $n = 12$)	0.409
	History of congestive HF	5.28 (95% CI: 4.35–6.41; $n = 785$)	5.39 (95% CI: 4.24–6.86; $n = 146$)	0.834
	History of arterial hypertension	5.00 (95% CI: 4.02–6.21; $n = 314$)	5.45 (95% CI: 4.49–6.62; $n = 617$)	0.274
	History of diabetes mellitus	5.46 (95% CI: 4.50–6.62; $n = 756$)	4.78 (95% CI: 3.77–6.06; $n = 175$)	0.154
	Prior vascular disease	5.30 (95% CI: 4.38–6.40; $n = 818$)	5.41 (95% CI: 4.11–7.12; $n = 113$)	0.850
	Prior MI	5.26 (95% CI: 4.35–6.35; $n = 864$)	6.00 (95% CI: 4.36–8.26; $n = 67$)	0.350

Abbreviations: CI, confidence interval; CNS, central nervous system; F1.2, prothrombin fragment 1 + 2; FEU, fibrinogen equivalent units; HF, heart failure; hs-CRP, high-sensitivity C-reactive protein; hs-IL-6, high-sensitivity interleukin-6; MI, myocardial infarction; TAT, thrombin–anti-thrombin III complex; TIA, transient ischemic attack.
[a]Adjusted for demographic covariates as given in ►**Table 1** by a linear model.

Supplementary Table S5 Mean biomarker levels at baseline by CHADS2 score adjusted for demographic

Biomarker	CHADS2 score	Low	Moderate	High	*p*-Value[a]
hs-CRP (mg/L)	CHADS2 score	2.29 (95% CI: 1.90–2.75; *n* = 228)	2.37 (95% CI: 2.06–2.71; *n* = 363)	2.62 (95% CI: 2.25–3.04; *n* = 359)	0.002
	CHADS2 VAS score	2.25 (95% CI: 1.75–2.89; *n* = 141)	2.23 (95% CI: 1.82–2.73; *n* = 218)	2.54 (95% CI: 2.26–2.85; *n* = 591)	0.001
D-dimer (mg/L FEU)	CHADS2 score	0.31 (95% CI: 0.28–0.36; *n* = 215)	0.32 (95% CI: 0.29–0.35; *n* = 347)	0.36 (95% CI: 0.32–0.40; *n* = 339)	<0.001
	CHADS2 VAS score	0.32 (95% CI: 0.27–0.38; *n* = 138)	0.31 (95% CI: 0.27–0.35; *n* = 208)	0.34 (95% CI: 0.32–0.37; *n* = 555)	<0.001
hs-IL-6 (pg/mL)	CHADS2 score	2.27 (95% CI: 2.04–2.52; *n* = 213)	2.48 (95% CI: 2.29–2.68; *n* = 342)	2.73 (95% CI: 2.51–2.97; *n* = 339)	<0.001
	CHADS2 VAS score	2.14 (95% CI: 1.86–2.47; *n* = 134)	2.41 (95% CI: 2.17–2.68; *n* = 202)	2.66 (95% CI: 2.48–2.84; *n* = 558)	<0.001
F1.2 (pmol/L)	CHADS2 score	168.03 (95% CI: 144.40–195.53; *n* = 228)	167.17 (95% CI: 148.99–187.56; *n* = 364)	167.65 (95% CI: 148.19–189.67; *n* = 358)	0.192
	CHADS2 VAS score	179.22 (95% CI: 145.64–220.53; *n* = 142)	156.79 (95% CI: 132.47–185.57; *n* = 219)	167.80 (95% CI: 152.28–184.91; *n* = 589)	0.015
TAT (μg/L)	CHADS2 score	4.90 (95% CI: 3.87–6.20;*n* = 224)	5.50 (95% CI: 4.44–6.82; *n* = 358)	5.38 (95% CI: 4.37–6.62; *n* = 349)	0.167
	CHADS2 VAS score	4.75 (95% CI: 3.60–6.28; *n* = 141)	5.23 (95% CI: 4.11–6.64; *n* = 215)	5.44 (95% CI: 4.47–6.64; *n* = 575)	0.085

Abbreviations: CHADS2, congestive heart failure, hypertension, age ≥ 75 years, diabetes mellitus, prior stroke, thromboembolism or transient ischemic attack; CHADS2 VAS, congestive heart failure, hypertension, age ≥ 75 years, diabetes mellitus, prior stroke, thromboembolism or TIA, vascular disease, age 65–74 years and sex; CI, confidence interval; F1.2, prothrombin fragment 1 + 2; FEU, fibrinogen equivalent units; hs-CRP, high-sensitivity C-reactive protein; hs-IL-6, high-sensitivity interleukin-6; TAT, thrombin–anti-thrombin III complex.
[a]Adjusted for demographic covariates as given in ►**Table 1** by a linear model.

Supplementary Table S6 Mean biomarker levels at baseline by type of AF adjusted for demographic covariates

Biomarker	First-diagnosed AF	Paroxysmal AF	Persistent AF	Long-standing persistent AF	*p*-Value[a]
hs-CRP (mg/L)	2.69 (95% CI: 2.30–3.16; *n* = 260)	2.63 (95% CI: 2.11–3.27; *n* = 138)	2.31 (95% CI: 2.05–2.60; *n* = 513)	1.86 (95% CI: 1.21–2.86; *n* = 34)	0.189
D-dimer (mg/L FEU)	0.43 (95% CI: 0.38–0.48; *n* = 244)	0.39 (95% CI: 0.34–0.45; *n* = 130)	0.28 (95% CI: 0.26–0.31; *n* = 492)	0.24 (95% CI: 0.17–0.32; *n* = 30)	<0.001
hs-IL-6 (pg/mL)	2.71 (95% CI: 2.48–2.97; *n* = 245)	2.96 (95% CI: 2.61–3.35; *n* = 131)	2.36 (95% CI: 2.21–2.52; *n* = 481)	2.03 (95% CI: 1.58–2.60; *n* = 33)	<0.001
F1.2 (pmol/L)	208.77 (95% CI: 183.03–238.12; *n* = 260)	185.80 (95% CI: 155.20–222.43; *n* = 137)	147.32 (95% CI: 133.59–162.45; *n* = 514)	120.92 (95% CI: 84.78–172.48; *n* = 34)	<0.001
TAT (μg/L)	5.75 (95% CI: 4.60–7.18; *n* = 257)	5.91 (95% CI: 4.60–7.61; *n* = 134)	4.76 (95% CI: 3.89–5.82; *n* = 502)	4.64 (95% CI: 3.09–6.96; *n* = 33)	0.003

Abbreviations: AF, atrial fibrillation; CI, confidence interval; F1.2, prothrombin fragment 1 + 2; FEU, fibrinogen equivalent units; hs-CRP, high-sensitivity C-reactive protein; hs-IL-6, high-sensitivity interleukin-6; TAT, thrombin–anti-thrombin III complex.
[a]Adjusted for demographic covariates as given in ►**Table 1** by a linear model.

Supplementary Table S7 Geometric means of biomarker measurements at baseline by cardioversion strategy and clinical events

Biomarker	Cardioversion strategy = early	Cardioversion strategy = delayed
hs-CRP (mg/L)	2.32 (95% CI: 2.04–2.65; n = 446)	2.35 (95% CI: 2.12–2.61; n = 504)
D-dimer (mg/L FEU)	0.36 (95% CI: 0.32–0.39; n = 421)	0.31 (95% CI: 0.29–0.34; n = 480)
hs-IL-6 (pg/mL)	2.70 (95% CI: 2.51–2.92; n = 418)	2.37 (95% CI: 2.22–2.53; n = 476)
F1.2 (pmol/L)	180.53 (95% CI: 162.91–200.04; n = 448)	149.34 (95% CI: 136.48–163.40; n = 502)
TAT (µg/L)	4.53 (95% CI: 4.05–5.07; n = 440)	3.73 (95% CI: 3.41–4.08; n = 491)
**Biomarker**	**Cardioversion success = no**	**Cardioversion success = yes**
hs-CRP (mg/L)	2.77 (95% CI: 2.19–3.50; n = 116)	2.23 (95% CI: 2.03–2.45; n = 689)
D-dimer (mg/L FEU)	0.32 (95% CI: 0.27–0.39; n = 105)	0.32 (95% CI: 0.30–0.34; n = 653)
hs-IL-6 (pg/mL)	2.71 (95% CI: 2.40–3.07; n = 109)	2.41 (95% CI: 2.27–2.56; n = 642)
F1.2 (pmol/L)	157.82 (95% CI: 130.42–190.96; n = 115)	155.07 (95% CI: 143.12–168.02; n = 687)
TAT (µg/L)	4.29 (95% CI: 3.50–5.26; n = 114)	3.87 (95% CI: 3.57–4.19; n = 671)
**Biomarker**	**Bleeding event = no**	**Bleeding event = yes**
hs-CRP (mg/L)	2.30 (95% CI: 2.10–2.51; n = 837)	2.68 (95% CI: 2.10–3.42; n = 113)
D-dimer (mg/L FEU)	0.33 (95% CI: 0.31–0.35; n = 794)	0.33 (95% CI: 0.28–0.39; n = 107)
hs-IL-6 (pg/mL)	2.49 (95% CI: 2.36–2.63; n = 790)	2.72 (95% CI: 2.39–3.10; n = 104)
F1.2 (pmol/L)	165.83 (95% CI: 153.94–178.63; n = 838)	145.65 (95% CI: 124.88–169.87; n = 112)
TAT (µg/L)	4.15 (95% CI: 3.84–4.48; n = 820)	3.67 (95% CI: 3.07–4.38; n = 111)

Abbreviations: CI, confidence interval; F1.2, prothrombin fragment 1 + 2; FEU, fibrinogen equivalent units; hs-CRP, high-sensitivity C-reactive protein; hs-IL-6, high-sensitivity interleukin-6; TAT, thrombin–anti-thrombin III complex.

Supplementary Table S8 Biomarker change from baseline–end of treatment (adjusted for biomarker baseline level and treatment duration)

Biomarker	Rivaroxaban	VKA	t-Statistic[a]	p-Value[a]
hs-CRP	−12.5% (95% CI: −19.9% to −4.4%; n = 481)	−17.9% (95% CI: −27.2% to −7.4%; n = 259)	0.827	0.408
D-dimer	−32.3% (95% CI: −36.2% to −28.2%; n = 448)	−37.7% (95% CI: −42.5% to −32.5%; n = 245)	1.629	0.104
hs-IL-6	−9.2% (95% CI: −14.2% to −3.8%; n = 417)	−9.8% (95% CI: −16.6% to −2.5%; n = 229)	0.144	0.886
F1.2	2.7% (95% CI: −5.6% to 11.7%; n = 481)	−53.0% (95% CI: −58.1% to −47.3%; n = 264)	10.803	<0.001
TAT	−28.0% (95% CI: −34.9% to −20.4%; n = 462)	−23.1% (95% CI: −32.8% to −12.0%; n = 258)	−0.763	0.446

Abbreviations: CI, confidence interval; F1.2, prothrombin fragment 1 + 2; hs-CRP, high-sensitivity C-reactive protein; hs-IL-6, high-sensitivity interleukin-6; TAT, thrombin–anti-thrombin III complex; VKA, vitamin-K antagonist.
[a] p-Values and t statistics are calculated from a linear model with the biomarker baseline level, treatment duration and treatment arm as independent variables. Consequently, the given mean changes are adjusted for treatment duration and biomarker baseline level.

Supplementary Table S9 Association of biomarker changes from baseline–end of treatment with clinical variables (adjusted for biomarker baseline level and treatment duration)

Biomarker	Factor	p-Value[a]	Subgroup	Rivaroxaban	VKA	t-Statistic[b]	p-Value[b]
hs-CRP	OAC taken prior to study	0.085	OAC taken prior to study = no	−14.5% (95% CI: −23.0 to −5.1%; n = 348)	−23.6% (95% CI: −33.9 to −11.8%; n = 180)	1.242	0.215
			OAC taken prior to study = yes	−7.4% (95% CI: −21.7 to 9.5%; n = 133)	−2.7% (95% CI: −21.8 to 21.1%; n = 79)	−0.350	0.726
	Cardioversion strategy	0.299	Cardioversion strategy = early	−17.9% (95% CI: −29.6 to −4.1%; n = 237)	−22.2% (95% CI: −35.6 to −5.9%;n = 127)	0.494	0.622
			Cardioversion strategy = delayed	−7.8% (95% CI: −19.9 to 6.1%; n = 244)	−12.2% (95% CI: −28.4 to 7.7%; n = 132)	0.450	0.653
	Cardioversion success	0.192	Cardioversion success = no	−1.6% (95% CI: −25.7 to 30.1%; n = 49)	2.4% (95% CI: −25.1 to 39.9%; n = 39)	−0.186	0.852
			Cardioversion success = yes	−10.6% (95% CI: −19.3 to −1.1%; n = 371)	−17.9% (95% CI: −29.0 to −5.2%; n = 184)	0.943	0.346
	History of congestive HF	0.721	History of congestive HF = no	−14.8% (95% CI: −22.7 to −6.1%; n = 399)	−15.3% (95% CI: −25.6 to −3.6%; n = 225)	0.077	0.938
			History of congestive HF = yes	−0.7% (95% CI: −19.9 to 23.1%; n = 82)	−32.9% (95% CI: −51.8 to −6.5%; n = 34)	1.950	0.052
	Type of AF	0.786	Type of AF = first-diagnosed	−5.5% (95% CI: −19.6 to 11.1%; n = 143)	−37.2% (95% CI: −51.0 to −19.6%; n = 61)	2.715	0.007
			Type of AF = paroxysmal	−11.5% (95% CI: −30.8 to 13.2%; n = 62)	−1.0% (95% CI: −25.6 to 31.6%; n = 46)	−0.583	0.560
			Type of AF = persistent	−15.3% (95% CI: −24.9 to −4.6%; n = 264)	−12.2% (95% CI: −25.7 to 3.6%; n = 137)	−0.343	0.731
			Type of AF = long-standing persistent	-26.3% (95% CI: −58.9 to 32.0%; n = 11)	−29.6% (95% CI: −60.7 to 26.1%; n = 11)	0.108	0.914
D-dimer	OAC taken prior to study	<0.001	OAC taken prior to study = no	-36.0% (95% CI: −40.3 to −31.3%; n = 317)	−42.6% (95% CI: −47.8 to −37.0%; n = 171)	1.832	0.067
			OAC taken prior to study = yes	−23.1% (95% CI: −31.1 to −14.2%; n = 131)	−23.4% (95% CI: −33.8 to −11.4%; n = 74)	0.045	0.964
	Cardioversion strategy	0.866	Cardioversion strategy = early	−31.1% (95% CI: −37.9 to −23.6%; n = 221)	−39.0% (95% CI: −46.3 to −30.8%; n = 116)	1.672	0.095
			Cardioversion strategy = delayed	−33.4% (95% CI: −39.3 to −26.9%; n = 227)	−36.5% (95% CI: −44.4 to −27.5%; n = 129)	0.665	0.506
	Cardioversion success	0.307	Cardioversion success = no	−32.7% (95% CI: −44.5 to −18.5%; n = 43)	−43.3% (95% CI: −54.0 to −30.1%; n = 36)	1.181	0.238
			Cardioversion success = yes	−30.7% (95% CI: −35.2 to −25.9%; n = 350)	−34.5% (95% CI: −40.5 to −27.9%; n = 172)	0.940	0.348
	History of congestive HF	0.117	History of congestive HF = no	−33.9% (95% CI: −38.1 to −29.5%; n = 376)	−37.7% (95% CI: −42.8 to −32.2%; n = 216)	1.079	0.281
			History of congestive HF = yes	−23.1% (95% CI: −33.6 to −10.8%; n = 72)	−37.4% (95% CI: −50.4 to −21.1%; n = 29)	1.486	0.138
	Type of AF	0.979	Type of AF = first-diagnosed	−33.2% (95% CI: −40.2 to −25.3%; n = 131)	−34.4% (95% CI: −44.5 to −22.3%; n = 56)	0.177	0.860
			Type of AF = paroxysmal	−32.1% (95% CI: −42.6 to −19.7%; n = 56)	−37.7% (95% CI: −48.6 to −24.7%; n = 43)	0.674	0.501
			Type of AF = persistent	−32.1% (95% CI: −37.3 to −26.5%; n=251)	−39.1% (95% CI: −45.5 to −32.0%; n = 131)	1.574	0.116
			Type of AF = long-standing persistent	−27.3% (95% CI: −52.1 to 10.4%; n = 9)	−32.7% (95% CI: −53.9 to −1.8%; n = 11)	0.272	0.785
hs-IL-6	OAC taken prior to study	0.061	OAC taken prior to study = no	−11.9% (95% CI: −17.7 to −5.8%; n = 304)	−11.8% (95% CI: −19.7 to −3.2%; n = 157)	−0.018	0.985
			OAC taken prior to study = yes	−1.4% (95% CI: −11.7 to 10.1%; n = 113)	−5.0% (95% CI: −17.4 to 9.2%; n = 72)	0.412	0.681
	Cardioversion strategy	0.343	Cardioversion strategy = early	−13.4% (95% CI: −21.8 to −4.2%; n = 201)	−10.4% (95% CI: −20.7 to 1.3%; n = 113)	−0.492	0.623
			Cardioversion strategy = delayed	−5.3% (95% CI: −13.6 to 3.7%; n = 216)	−8.7% (95% CI: −19.8 to 4.0%; n = 116)	0.512	0.609
	Cardioversion success	0.074	Cardioversion success = no	6.5% (95% CI: −11.4 to 28.0%; n = 41)	4.9% (95% CI: −14.7 to 29.0%; n = 32)	0.107	0.915
			Cardioversion success = yes	−6.9% (95% CI: −12.8 to −0.7%; n = 322)	−8.3% (95% CI: −16.4 to 0.5%; n = 163)	0.264	0.792
	History of congestive HF	0.111	History of congestive HF = no	−11.4% (95% CI: −16.9 to −5.6%; n = 346)	−9.8% (95% CI: −17.0 to −2.0%; n = 201)	−0.341	0.733
			History of congestive HF = yes	2.9% (95% CI: −10.7 to 18.5%; n = 71)	−9.6% (95% CI: −27.7 to 13.0%;n = 28)	0.967	0.334

Supplementary Table S9 (*Continued*)

Biomarker	Factor	*p*-Value[a]	Subgroup	Rivaroxaban	VKA	*t*-Statistic[b]	*p*-Value[b]
	Type of AF	0.561	Type of AF = first-diagnosed	−6.0% (95% CI: −15.2 to 4.3%; *n* = 129)	−19.7% (95% CI: −32.0 to −5.1%; *n* = 50)	1.578	0.115
			Type of AF = paroxysmal	−7.6% (95% CI: −21.4 to 8.6%; *n* = 54)	1.3% (95% CI: −15.0 to 20.7%; *n* = 45)	−0.759	0.448
			Type of AF = persistent	−10.9% (95% CI: −17.7 to −3.6%; *n* = 223)	−7.6% (95% CI: −17.1 to 2.9%; *n* = 120)	−0.534	0.594
			Type of AF = long-standing persistent	−14.1% (95% CI: −40.8 to 24.7%; *n* = 10)	−18.4% (95% CI: −42.8 to 16.5%; *n* = 11)	0.195	0.846
F1.2	OAC taken prior to study	0.002	OAC taken prior to study = no	−1.8% (95% CI: −11.2 to 8.5%; *n* = 347)	−58.7% (95% CI: −63.9 to −52.6%; *n* = 183)	10.115	<0.001
			OAC taken prior to study = yes	15.0% (95% CI: −2.4 to 35.4%; *n* = 134)	−36.8% (95% CI: −48.8 to −22.1%; *n* = 81)	4.560	<0.001
	Cardioversion strategy	0.343	Cardioversion strategy = early	−13.4% (95% CI = (−21.8 to −4.2%; *n* = 201)	−10.4% (95% CI: −20.7 to 1.3%; *n* = 113)	−0.492	0.623
			Cardioversion strategy = delayed	−5.3% (95% CI: −13.6 to 3.7%; *n* = 216)	−8.7% (95% CI: −19.8 to 4.0%; *n* = 116)	0.512	0.609
	Cardioversion success	0.074	Cardioversion success = no	6.5% (95% CI: −11.4 to 28.0%; *n* = 41)	4.9% (95% CI: −14.7 to 29.0%; *n* = 32)	0.107	0.915
			Cardioversion success = yes	−6.9% (95% CI: −12.8 to −0.7%; *n* = 322)	−8.3% (95% CI: −16.4 to 0.5%; *n* = 163)	0.264	0.792
	History of congestive HF	0.033	History of congestive HF = no	1.4% (95% CI: −7.5 to 11.1%; *n* = 400)	−55.8% (95% CI: −60.9 to −50.1%; *n* = 231)	10.687	<0.001
			History of congestive HF = yes	9.6% (95% CI: −10.6 to 34.3%; *n* = 81)	−27.6% (95% CI: −47.3 to −0.3%; *n* = 33)	2.145	0.032
	Type of AF	0.754	Type of AF = first-diagnosed	11.7% (95% CI: −4.4 to 30.5%; *n* = 142)	−53.3% (95% CI: −63.1 to −40.7%; *n* = 61)	6.047	<0.001
			Type of AF = paroxysmal	0.6% (95% CI: −20.4 to 27.3%; *n* = 62)	−47.0% (95% CI: −59.8 to −30.3%; *n* = 45)	3.490	0.001
			Type of AF = persistent	−0.5% (95% CI: −11.3 to 11.5%; *n* = 265)	−54.5% (95% CI: −61.1 to −46.9%; *n* = 142)	7.917	<0.001
			Type of AF = long-standing persistent	−20.8% (95% CI: −54.6 to 38.2%; *n* = 11)	−46.3% (95% CI: −68.5 to −8.4%; *n* = 12)	0.989	0.323
TAT	OAC taken prior to study	0.655	OAC taken prior to study = no	−27.4% (95% CI: −35.5 to −18.3%; *n* = 335)	−26.7% (95% CI: −37.6 to −13.9%; *n* = 179)	−0.099	0.921
			OAC taken prior to study = yes	−29.5% (95% CI: −41.8 to −14.6%; *n* = 127)	−14.1% (95% CI: −32.8 to 9.7%; *n* = 79)	−1.250	0.212
	Cardioversion strategy	0.805	Cardioversion strategy = early	−26.4% (95% CI: −38.2 to −12.4%; *n* = 232)	−22.9% (95% CI: −37.7 to −4.6%; *n* = 124)	−0.381	0.704
			Cardioversion strategy = delayed	−29.4% (95% CI: −39.9 to −17.0%; *n* = 230)	−23.6% (95% CI: −38.9 to −4.3%; *n* = 134)	−0.650	0.516
	Cardioversion success	0.717	Cardioversion success = no	−18.5% (95% CI: −40.7 to 12.1%;*n* = 47)	−19.0% (95% CI: −42.9 to 14.8%;*n* = 39)	0.027	0.979
			Cardioversion success = yes	−26.2% (95% CI: −34.2 to −17.2%; *n* = 359)	−17.3% (95% CI: −29.7 to −2.6%; *n* = 180)	−1.125	0.261
	History of congestive HF	0.787	History of congestive HF = no	−27.7% (95% CI: −35.3 to −19.3%; *n* = 384)	−22.6% (95% CI: −33.0 to −10.7%; *n* = 228)	−0.735	0.462
			History of congestive HF = yes	−29.3% (95% CI: −44.6 to −9.8%; *n* = 78)	−26.7% (95% CI: −50.5 to 8.7%; *n* = 30)	−0.154	0.878
	Type of AF	0.398	Type of AF = first-diagnosed	−17.1% (95% CI: −30.9 to −0.5%; *n* = 141)	−17.5% (95% CI: −37.6 to 9.0%; *n* = 60)	0.029	0.977
			Type of AF = paroxysmal	−33.3% (95% CI: −49.6 to −11.6%; *n* = 59)	−17.1% (95% CI: −39.6 to 13.9%; *n* = 46)	−1.007	0.314
			Type of AF = persistent	−31.7% (95% CI: −40.4 to −21.7%; *n* = 250)	−29.9% (95% CI: −41.8 to −15.7%; *n* = 137)	−0.220	0.826
			Type of AF = long-standing persistent	−43.2% (95% CI: −70.4 to 8.7%; *n* = 11)	17.0% (95% CI: −38.9 to 124.1%; *n* = 11)	−1.546	0.123

Abbreviations: AF, atrial fibrillation; ANCOVA, analysis of covariance; CI, confidence interval; F1.2, prothrombin fragment 1+2; HF, heart failure; hs-CRP, high-sensitivity C-reactive protein; hs-IL-6, high-sensitivity interleukin-6; OAC, oral anticoagulant; TAT, thrombin–anti-thrombin III complex; VKA, vitamin K antagonist.

[a]*p*-Values were calculated from an ANCOVA that contained the biomarker baseline level, treatment duration and the treatment arm in addition to the respective factor variable.

[b]*p*-Values and *t*-statistics were calculated from a linear model with the biomarker baseline level, treatment duration, treatment arm and the respective subgroup variable (as a main effect and in interaction with treatment) as independent variables. Consequently, the given mean changes are adjusted for treatment duration and biomarker baseline level.
